# Efficacy and safety of once daily versus twice daily mesalazine for mild-to-moderate ulcerative colitis: A meta-analysis of randomized controlled trials

**DOI:** 10.1097/MD.0000000000015113

**Published:** 2019-04-05

**Authors:** Xin Zheng, Zhen Zhang, Botao Wang, Jiaxin Li, Chongyang Qiu, Qi Zhang, Ximo Wang

**Affiliations:** a Graduate School of Tianjin Medical University; b Tianjin Institute of Acute Abdominal Disease of Integrated Traditional Chinese and Western Medicine; c Tianjin Hospital of Integrated Traditional Chinese and Western Medicine, Tianjin; d Peking University People's Hospital, Beijing, China.

**Keywords:** mesalazine, meta-analysis, once daily, twice daily, ulcerative colitis

## Abstract

**Background:**

We aimed to conduct a meta-analysis to evaluate the efficacy and safety between once daily (OD) and twice daily (BD) regime dosing of mesalazine for mild-to-moderate ulcerative colitis (UC).

**Methods:**

PubMed, Embase, the Cochrane library, and Web of Science from 1990 to November 2018 were investigated. We searched randomized controlled trials (RCTs) comparing OD with BD regime dosing of mesalazine for mild-to-moderate UC. The software Review Manager 5.3 was used to pool the risk ratio (RR) with a 95% confidence interval (CI).

**Results:**

Eight RCTs containing 3495 patients were identified. Regardless of induction of remission or maintenance of remission of UC, OD regime dosing of mesalazine was as effective as BD regime dosing in clinical and endoscopic remission and clinical remission. Also, no obvious difference was found between OD and BD regime dosing of mesalazine regardless of total adverse events, treatment-related adverse events or serious adverse events.

**Conclusion:**

OD is as effective and safe as BD regime dosing of mesalazine for active UC.

## 1 Introduction

Ulcerative colitis (UC) is a chronic inflammatory disease involving the mucosa of colorectum, characterized by recurrent remission and relapse.^[1]^ The incidence of ulcerative colitis stabilizes in high-incidence areas such as northern Europe and North America. For instance, incidence rates for ulcerative colitis range from 2.2 to 14.3 cases per 100,000 persons per year in North America.^[2]^

Mesalazine remains the first-line drug for mild-to-moderate UC.^[3]^ In the 1940s, the practice of conventional dosing schedule began with sulfasalazine, and a minimum of twice daily (BD) mesalazine was used because of it reducing the toxicity and side effects.^[4]^ However, a divided-dosing regime might lead to noncompliance.^[5]^ Many researches have revealed that new kinds of mesalazine (such as pH-dependent formulations, time-dependent formulations, and multimatrix system) can be administered once daily (OD).^[6–13]^ It was convenient and simple dosing regime for patients.

Although three reviews^[14–16]^ assessed the efficacy and safety between OD and multiple daily mesalazine, no meta-analysis was found to evaluate the efficacy and safety between OD regime dosing of mesalazine and BD regime. Therefore, we performed a meta-analysis to compare the efficacy and adverse events (AEs) of OD regime dosing of mesalazine with BD for UC.

## 2 Methods

Ethical approval or patient consent is not required because the present study is a review of previously published articles.

### 2.1 Search strategy

Randomized controlled trials (RCTs) published were searched in English. PubMed, Embase, the Cochrane library, and Web of Science from 1990 to November 2018 were investigated by 2 authors. The keywords included “mesalazine,” “mesalamine,” “5-aminosalicylic acid,” “5-ASA,” “Inflammatory bowel disease,” “ulcerative colitis,” “once daily,” “twice daily,” and “randomized controlled trials,” which were combined with any derivatives of these terms. What is more, manual searching of reference lists from existing review articles and identified additional studies was also performed.

### 2.2 Inclusion and exclusion criteria

The inclusion criteria included that: the study covered published clinical RCTs comparing OD with BD mesalazine for mild-to-moderate UC, eligible patients were 18 years of age or older, with a confirmed clinical, endoscopic, and histological diagnosis of UC, and the outcomes must include one of clinical and endoscopic remission, clinical remission, mucosal healing, and adverse events. Studies without full-text and sufficient information were excluded.

### 2.3 Extraction of data and assessment of outcomes

The extracted data included: author, year and country of publication, the number of patients randomized and intention-to-treat (ITT), characteristics of the patients (age and gender), interventions, duration of therapy, inclusion criteria, and criteria to define remission.

The primary outcome of articles included was clinical and endoscopic remission of OD compared with BD mesalazine. The secondary outcomes included clinical remission, and adverse events (total adverse events, treatment-related adverse events, and serious adverse events).

### 2.4 Assessment of risk of bias

All RCTs were evaluated for the risk of bias with the Cochrane Collaboration's Risk of Bias Tool.^[17]^ We assessed the risk of bias of RCTs using the following items: random sequence generation, allocation concealment, blinding of participants and personnel, blinding of outcome assessment, incomplete outcome data, selective reporting, and other bias. Every parameter was classified into 3 categories (low risk, high risk, and unclear risk). The risk of bias of those studies was presented in Figure 2.

### 2.5 Statistical analysis

We carried out the meta-analysis with Review Manager 5.3. The risk ratio (RR) with a 95% confidence interval (CI) was presented. The Cochrane Handbook's *Q* test and I2 statistics were used to evaluate the heterogeneity among these eligible literatures.^[18]^ If there was no significant heterogeneity (*P≥*.05 and I2≤50%), the fixed-effects model was applied. Else, the random-effects model was used. The efficacy was analyzed in patients valid for ITT. All statistical tests were 2 sided, and values of *P < *.05 were considered as statistical significance.

## 3 Results

### 3.1 Description of the studies included

**Figure 1:**
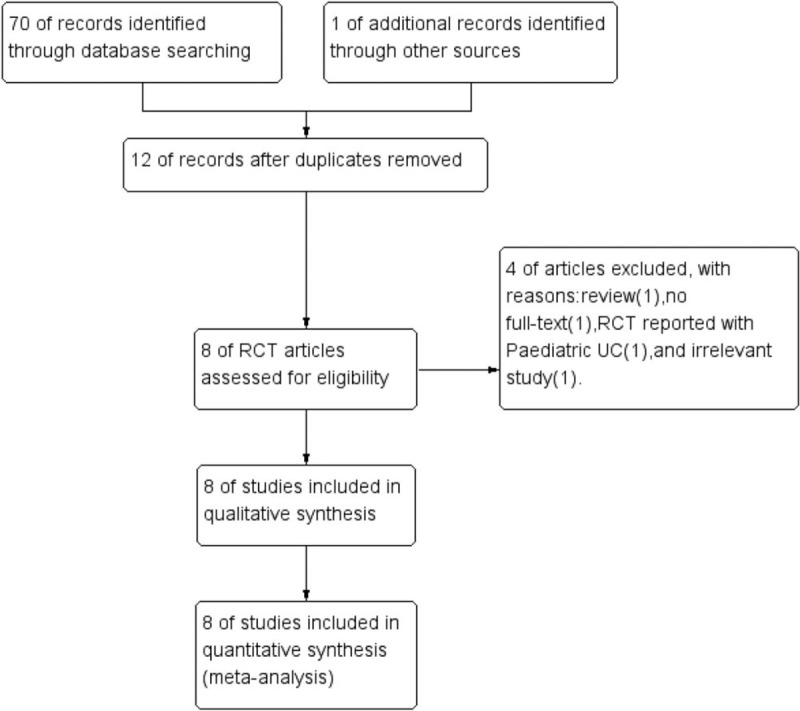
Flowchart of literature search.

Flowchart of the search strategy is shown in Figure 1. Seventy-one studies were identified through database searching and other sources. Of 71 records screened, 8 RCTs^[6–13]^ were eligible.

**Figure 2:**
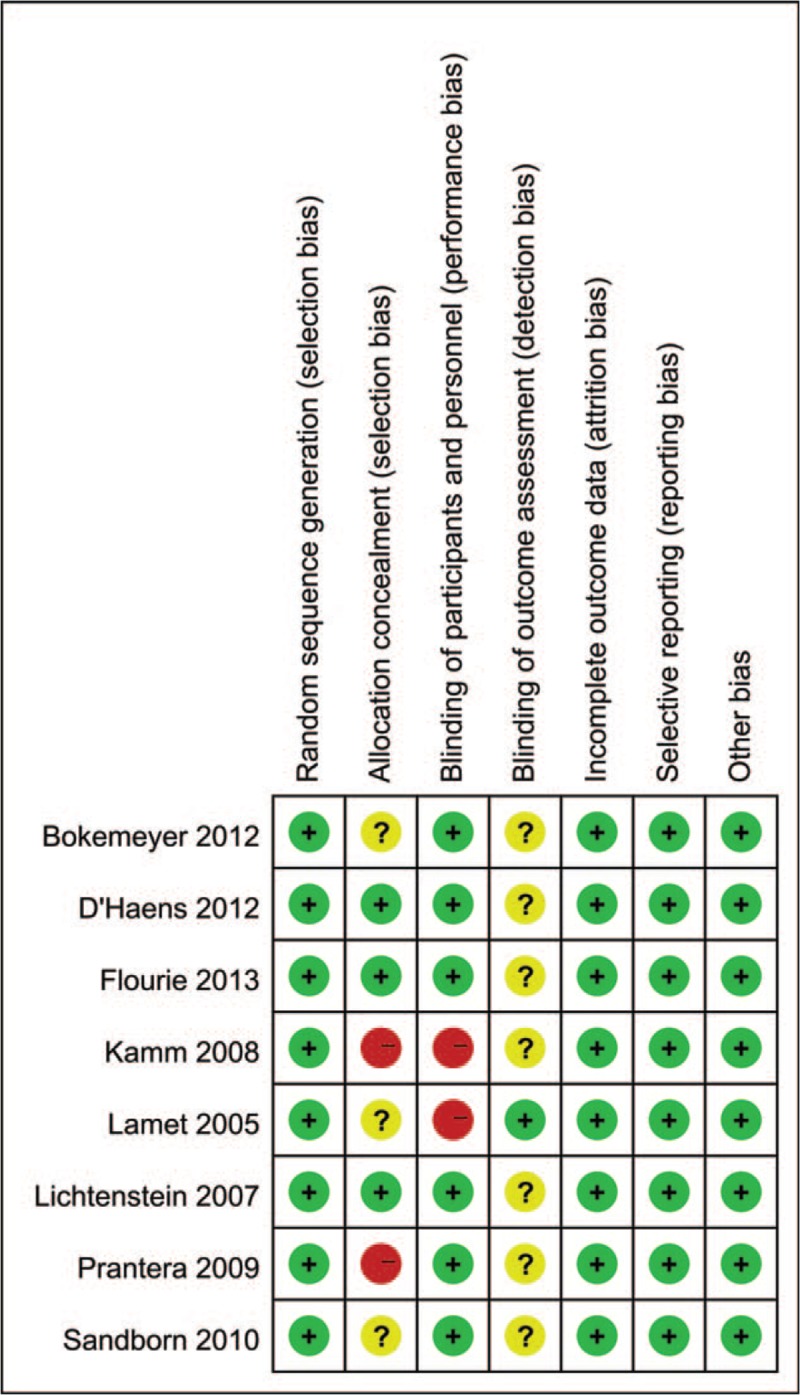
Risk of bias summary. This risk of bias tool incorporates the assessment of randomization, allocation concealment, blinding of participants and personnel, blinding of outcome assessment, incomplete outcome data, selective reporting, and other bias. The items were judged as “low risk” “unclear risk,” or “high risk”. Green means “low risk,” yellow means “unclear risk,” and red means “high risk.”

Table 1 shows characteristics of the 8 studies included. The 8 studies included 3495 patients randomized, of whom 1731 (49.5%) in OD mesalazine group and 1764 (50.5%) in BD mesalazine group. The number of patients valid for ITT was 3375, of whom 1667 (49.4%) in OD mesalazine group and 1708 (50.6%) in BD mesalazine group.

**Table 1:**
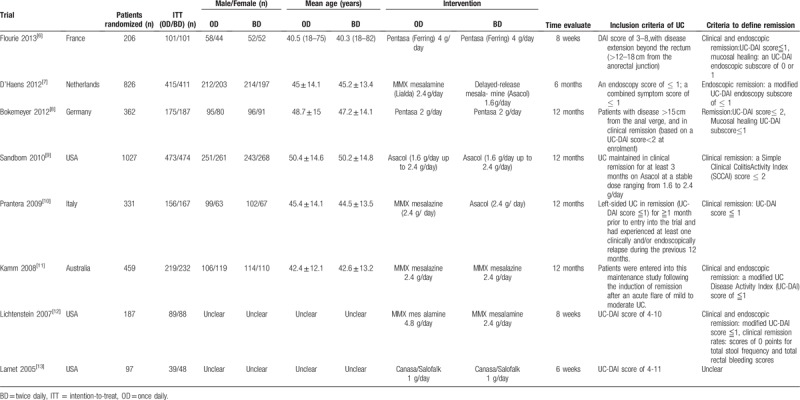
Characteristics of studies included.

### 3.2 Induction of remission

#### 3.2.1 Clinical and endoscopic remission

**Figure 3:**
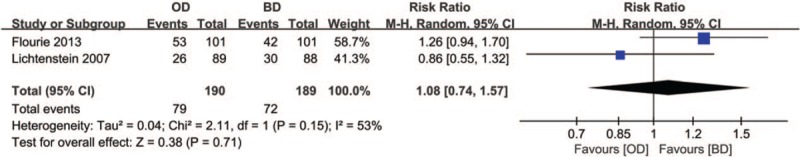
Forest plot of RCTs of once daily versus twice daily mesalazine on clinical and endoscopic remission for induction of remission of ulcerative colitis. BD = twice daily, OD = once daily, RCTs = randomized controlled trials.

Two RCTs^[6,12]^ representing 379 patients (OD group, n = 190; BD group, n = 189) reported clinical and endoscopic remission for induction of remission of UC. The random-effects model was applied because of significant heterogeneity (I^2^ = 53%) among the studies revealed by a heterogeneity test. A pooled analysis implied that there was no significant difference in clinical and endoscopic remission rates between the two treatment groups (RR = 1.08, 95% CI: 0.74–1.57, *P* = .71) (Fig. 3).

#### 3.2.2 Clinical remission

**Figure 4:**
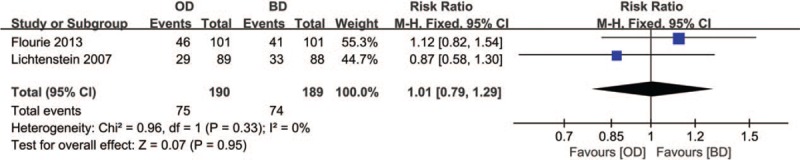
Forest plot of RCTs of once daily versus twice daily mesalazine on clinical remission for induction of remission of ulcerative colitis. BD = twice daily, OD = once daily, RCTs = randomized controlled trials.

Of 8 studies, 2 RCTs^[6,12]^ representing 379 patients described clinical remission. Because of no heterogeneity (*P* = .33, *I*^2^ = 0%) between 2 studies, the fixed-effects model was used. No notable difference was observed between the 2 groups (RR = 1.01, 95% CI: 0.79–1.29, *P* = .95) (Fig. 4).

#### 3.2.3 Adverse events

**Figure 5:**
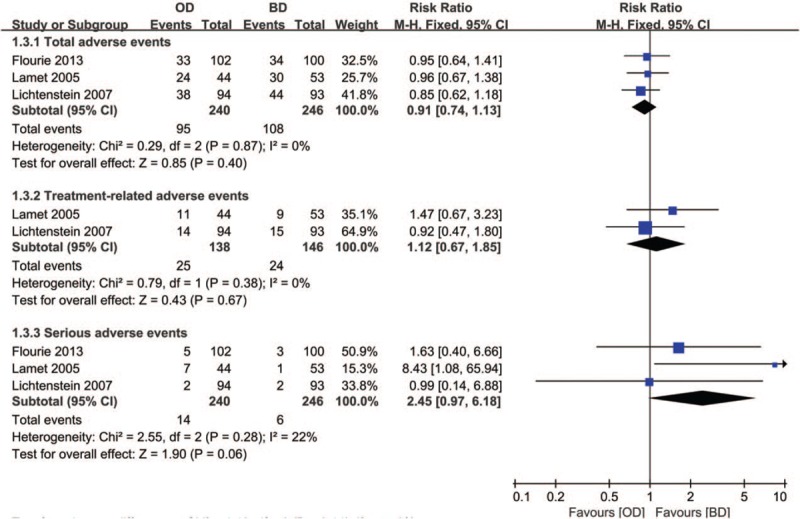
Forest plot of RCTs of once daily versus twice daily mesalazine on adverse events for induction of remission of ulcerative colitis. BD = twice daily, OD = once daily, RCTs = randomized controlled trials.

Adverse events were described in 3 RCTs.^[6,12,13]^ A total of 486 patients (OD group, n = 240; BD group, n = 246) were identified. There was no significant difference between the 2 groups in the incidence of total AEs (3 RCTs,^[6,12,13]^ n = 586, RR = 0.91, 95% CI: 0.74–1.13, *P* = .4, *I*^2^ = 0; Fig. 5), treatment-related AEs (2 RCTs,^[12,13]^ n = 284, RR = 1.12, 95% CI: 0.67–1.85, *P* = .67, *I*^2^ = 0; Fig. 5), and serious AEs (3 RCTs,^[6,12,13]^ n = 486, RR = 2.45, 95% CI: 0.97–6.18, *P* = .06, *I*^2^ = 22%; Fig. 5).

### 3.3 Maintenance of remission

#### 3.3.1 Clinical and endoscopic remission

**Figure 6:**
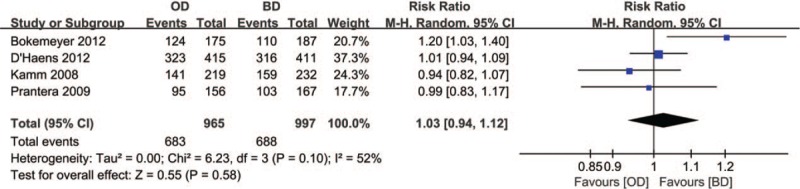
Forest plot of RCTs of once daily versus twice daily mesalazine on clinical and endoscopic remission for maintenance of remission of ulcerative colitis. BD = twice daily, OD = once daily, RCTs = randomized controlled trials.

Four RCTs^[7,8,10,11]^ reported clinical and endoscopic remission. A total of 1962 patients (OD group, n = 965; BD group, n = 997) were identified 683 (70.8%) of patients in OD group and 688 (69.0%) of patients in BD group achieved clinical and endoscopic remission. The random-effects model was applied because of moderate heterogeneity (*I*^2^ = 52%) among the studies. There was no notable difference in clinical and endoscopic remission rate between OD and BD mesalazine group (RR = 1.03, 95% CI: 0.94–1.12, *P* = .58) (Fig. 6).

#### 3.3.2 Clinical remission

**Figure 7:**
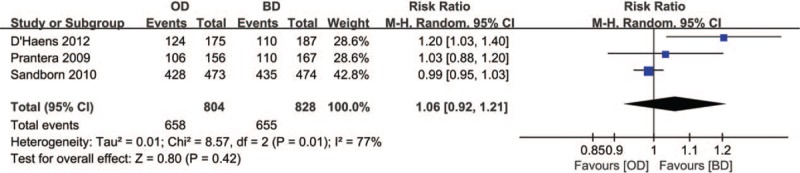
Forest plot of RCTs of once daily versus twice daily mesalazine on clinical remission for maintenance of remission of ulcerative colitis. BD = twice daily, OD = once daily.

Clinical remission was described in 3 RCTs. A total of 1632 patients (OD group, n = 804; BD group, n = 828) were identified, and 658 (81.8%) of patients in OD group and 655 (79.1%) of patients in BD group achieved clinical remission. Because significant heterogeneity (*P* = .01, *I*^2^ = 77%) was detected among these studies, the random-effects model was applied. No obvious difference was observed between the two groups with respect to clinical remission (RR = 1.06, 95% CI: 0.92–1.21, *P* = .42) (Fig. 7).

#### 3.3.3 Adverse events

**Figure 8:**
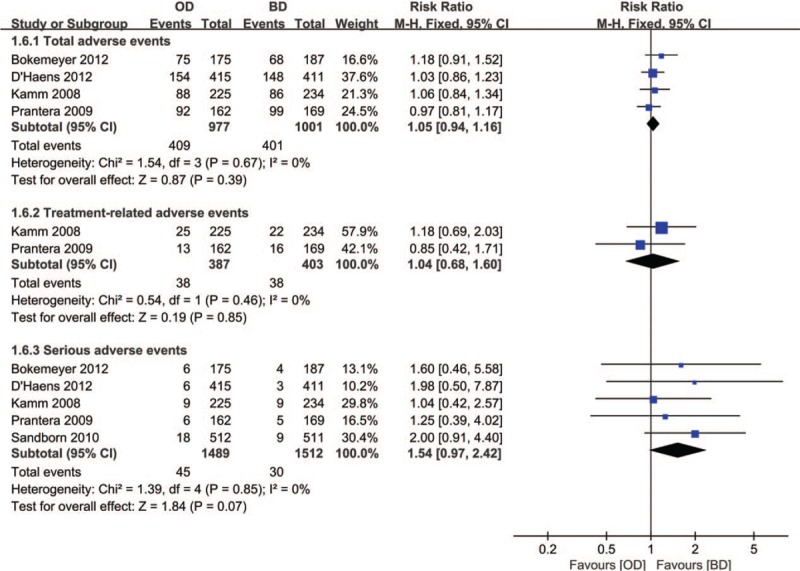
Forest plot of RCTs of once daily versus twice daily mesalazine on adverse events for maintenance of remission of ulcerative colitis. BD = twice daily, OD = once daily, RCTs = randomized controlled trials.

Five studies included reported adverse events. There was no significant difference between the two groups in the incidence of total AEs (4 RCTs^[7,8,10,11]^, n = 1978, RR = 1.05, 95% CI: 0.94–1.16, *P* = .39, I^2^ = 0; Fig. 8), treatment-related AEs (2 RCTs,^[10,11]^ n = 790, RR = 1.04, 95% CI: 0.68–1.60, *P* = .85, I^2^ = 0; Fig. 8), and serious AEs (5 RCTs,^[7–11]^ n = 3001, RR = 1.54, 95% CI: 0.97–2.42, *P* = .06, *I*^2^ = 0; Fig. 8).

## 4 Discussion

Three meta-analyses^[14–16]^ have assessed the efficacy and safety of OD compared with multiple daily regime dosing of mesalazine, but no meta-analysis to evaluate these between OD and BD regime dosing of mesalazine was found. So, this meta-analysis was performed to evaluate the efficacy and safety of OD compared with BD regime dosing of mesalazine for mild-to-moderate UC. The results implied that OD was as effective and safe as BD regime dosing of mesalazine for induction and maintenance of remission of UC.

With regard to the efficacy between the 2 treatments, OD regime dosing of mesalazine was as effective as BD regime dosing in clinical and endoscopic remission (induction of remission, OD vs BD, 41.6% vs 38.1%, *P* = .71; maintenance of remission, OD vs BD, 70.8% vs 69%, *P* = .58) for UC. Similar results were found with regard to the clinical remission (induction of remission, OD vs BD, 39.5% vs 39.2%, *P* = .95; maintenance of remission, OD vs BD, 81.8% vs 79.1%, *P* = .42). Regardless of clinical and endoscopic remission or clinical remission, it seemed that OD regime dosing of mesalazine was more effective than BD regime dosing, but no notable difference was found.

In terms of safety, OD regime dosing of mesalazine was as safe as BD regime dosing regardless of total AEs, treatment-related AEs or serious AEs because of no significant difference between the two groups in the incidence of total AEs, treatment-related AEs, and serious AEs.

Newer mesalazine formulations with luminal release properties could reduce the toxicity and improve the curative effect.^[19,20]^ The same efficacy and safety between OD and BD regime dosing may be associated with the characteristics of newer mesalazine formulations.

Although no difference between the 2 groups, taking less frequent dosing could improve compliance to treatment for UC.^[21,22]^ Studies about compliance are needed to compare OD to BD regime dosing of mesalazine for UC in the future.

Some limitations of this meta-analysis were described as following. Of 8 studies included, 7 studies did not report blinding of outcome assessment, increasing the detection bias of the meta-analysis. In addition, the drug dosage among various studies was inconsistent. These limitations may increase the risk of bias of this meta-analysis.

In conclusion, OD is as effective and safe as BD regime dosing of mesalazine for mild-to-moderate UC. And OD regime dosing of mesalazine is more convenient than BD regime because patients need long-term medication for UC.

## Author contributions

**Conceptualization:** Jiaxin Li, Ximo Wang.

**Data curation:** Xin Zheng, Ximo Wang.

**Formal analysis:** Xin Zheng, Zhen Zhang, Botao Wang.

**Investigation:** Zhen Zhang, Jiaxin Li.

**Methodology:** Botao Wang.

**Project administration:** Ximo Wang.

**Resources:** Ximo Wang.

**Software:** Xin Zheng, Zhen Zhang, Jiaxin Li, Chongyang Qiu.

**Supervision:** Botao Wang, Jiaxin Li, Chongyang Qiu, Qi Zhang.

**Validation:** Xin Zheng, Zhen Zhang, Botao Wang, Qi Zhang.

**Writing – Original Draft:** Xin Zheng.

**Writing – Review & Editing:** Ximo Wang.
